# Solubility Improvement of Benexate through Salt Formation Using Artificial Sweetener

**DOI:** 10.3390/pharmaceutics10020064

**Published:** 2018-05-26

**Authors:** Okky Dwichandra Putra, Daiki Umeda, Eriko Fujita, Tamami Haraguchi, Takahiro Uchida, Etsuo Yonemochi, Hidehiro Uekusa

**Affiliations:** 1 School of Pharmacy and Pharmaceutical Sciences, Hoshi University, 2-4-41 Ebara, Shinagawa, Tokyo 142-8501, Japan; okky.putra@astrazeneca.com (O.D.P.); d.a.i.k.i102475@gmail.com (D.U.); s131207@hoshi.ac.jp (E.F.); 2 Department of Chemistry, Tokyo Institute of Technology, 2-12-1 Ookayama, Meguro, Tokyo 152-8551, Japan; 3 Faculty of Pharmaceutical Sciences, Mukogawa Women’s University, 11-68 Koshien 9-Bancho, Nishinomiya, Hyogo 663-8179, Japan; tamami@mukogawa-u.ac.jp (T.H.); takahiro@mukogawa-u.ac.jp (T.U.)

**Keywords:** benexate, crystal engineering, solubility, dissolution, sweetener

## Abstract

Benexate, a drug used clinically as a defensive type anti-ulcer agent, has poor solubility and a bitter taste. To improve its solubility, a crystal engineering approach was proposed with the formation of novel salts using an artificial sweetener as a salt co-former. This was also expected to address the bitter taste of the drug. In this work, we report on the preparation and evaluation of the physicochemical properties of the novel salts benexate saccharinate monohydrate and benexate cyclamate whose crystal structures were determined by single-crystal X-ray structure analysis. These novel salts showed higher solubility and faster dissolution profiles that were associated with the occurrence of local layered-like structures. They also showed better moisture uptake profiles and were classified as non-hygroscopic materials. Therefore, benexate saccharinate monohydrate and benexate cyclamate expedited the development of sweet pharmaceutical salts of benexate with improved performances.

## 1. Introduction

The pharmaceutical field has recently shown enormous interest in improving the solubilities and dissolution rates of poorly soluble drugs. Many techniques for the same have been widely reported in the literature, including solid dispersion, particle size reduction, development of various nanoparticulate delivery systems, and complexation with cyclodextrin derivatives [1,2,3,4,5,6,7,8]. However, these techniques have some general limitations. The use of an amorphous system in solid dispersion improves solubility by taking advantage of a thermodynamically unstable system. However, an amorphous solid in solid dispersion may reorder to become crystalline, which would influence the performance of the pharmaceutical dosage form [9]. In addition, particle size reduction is reported to have limitations when the drug is compressed into a tablet [10]. The preparation of nanoparticulate delivery is a cutting-edge technique to improve the bioavailability of poorly soluble drug materials. However, some concerns regarding the safety of this technique have been addressed recently [11,12]. An inclusion complex with cyclodextrin derivatives is a popular technique to improve the solubility of poorly soluble drugs. However, this technique faces some challenges when a drug is not able to form a strong interaction within the cavity, resulting in insignificant solubility improvement [13].

An alternative technique for overcoming the solubility challenge of poorly soluble drugs without changing the pharmacophore structure of the drug is to develop new crystalline forms. Polymorphs, solvates, salts, and cocrystals are popular and widely accepted in crystal engineering [14,15,16,17,18]. Other than for solubility improvements, the preparation of new crystalline forms has also been reported in the exploration of physicochemical properties of drug materials, i.e., poor tabletability, chemical instability, hygroscopicity, etc. [18,19,20,21,22,23]. Pharmaceutical industries use this technique in the context of the pharmacological effect in fixed-dose combination drugs, as illustrated in valsartan-sacubitril, inosine pranobex, and dimenhydrinate [24,25,26,27,28,29].

Among the above-mentioned four new crystalline forms, the pharmaceutical salts usually show more solubility and a better dissolution profile than the free forms of drugs owing to the former’s dissociation capability in the solvent [30]. To tackle sub-optimal physicochemical properties, by pairing a drug molecule with its counterion, more than 50% of drugs are administrated as salts [31,32]. A common strategy to produce pharmaceutical salts utilizes the so-called p*K*_a_ rule. This rule states that to accomplish salt formation, instead of a neutral cocrystal, the required p*K*_a_ difference between a base and an acid is at least three units, especially when the drug substance is either a weak acid or a weak base [32]. Exceptions may be found when a salt has acceptable stability, despite a small difference in the p*K*_a_ value [33]. 

**Scheme 1:**
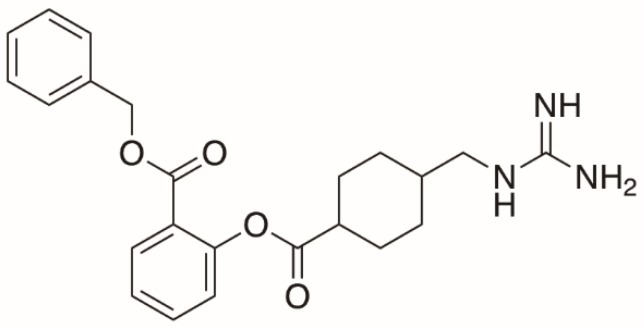
The molecular structure of benexate (BEX).

In this study, we explored benexate (BEX, Scheme 1), which is used clinically as a defensive type anti-ulcer agent. Its defensive effects on the gastric mucosa have been demonstrated in the promotion of prostaglandin synthesis, protein secretion, and an improved blood flow to the gastrointestinal tract [34]. This drug is marketed as benexate hydrochloride (BEX–HCl) salt and is inclusion complexed with β-cyclodextrin. The hydrochloride salt was originally made to improve the solubility of BEX, which was not significant owing to the high molecular weight of BEX–HCl (molecular weight = 445.93 g/mol). Later, BEX–HCl was complexed with β-cyclodextrin to improve its solubility and to reduce the bitter taste of this drug [35,36]. 

From the crystal engineering perspective, BEX is both an interesting and challenging compound because it has two major problems: a low solubility and a bitter taste. To date, no new salt structure has been reported that could tackle the unfavorable physicochemical property issues associated with BEX. Therefore, we attempted to explore artificial sweeteners as salt co-formers with the purpose of naturally overcoming the native unpleasant taste of BEX. Artificial sweeteners have been reported to improve the solubility and dissolution rate of several drugs, as illustrated with quinine, haloperidol, mirtazapine, pseudoephedrine, lamivudine, risperidone, sertraline, venlafaxine, zolpidem, amlodipine, and piroxicam [37,38]. This implies that artificial sweeteners are also potentially applicable to BEX’s low solubility issue. 

## 2. Materials and Methods 

### 2.1. Materials

BEX-HCl pharmaceutical grade was donated by Nagase Co. Ltd. (Osaka, Japan). Sodium saccharinate and sodium cyclamate (analytical grade) were purchased from Tokyo Chemical Industry (Tokyo, Japan). These compounds were used as received for all processes. Other chemicals used in this study were purchased from Nacalai Tesque (Tokyo, Japan) and used without further purification. 

### 2.2. Methods

#### 2.2.1. Preparation of Salts

New saccharinate and cyclamate salts of BEX were prepared by an anion exchange reaction using sodium saccharinate and sodium cyclamate in aqueous solution. BEX–HCl (2.229 g, 5 mmol) was dissolved in 50 mL ethanol:water (1:1) mixture in a 100 mL Erlenmeyer flask. Equimolar sodium saccharinate dihydrate (1.206 g, 5 mmol) was added to the solution and heated with stirring (60 °C, 50 rpm) until a clear solution was obtained. The resulting solution was cooled and left undisturbed at ambient temperature for two days to one week to yield colorless block-shaped crystals, which were suitable for single-crystal X-ray diffraction. The crystals were harvested by filtration. The same procedure was applied for the preparation of cyclamate salts of BEX and colorless-blocked shaped crystals were obtained after two weeks.

#### 2.2.2. Powder X-Ray Diffraction

Powder X-ray diffraction (PXRD) measurements were performed using a SMART-LAB X-ray diffractometer (Rigaku, Japan). The corresponding PXRD patterns were collected in reflection mode for 2θ = 5–40° at 25 °C with a step of 0.01° and a scan speed of 20° min^−1^ (Cu-*K*α source, 45 kV, 200 mA).

#### 2.2.3. Differential Scanning Calorimetry and Thermogravimetry

Differential scanning calorimetry (DSC) measurements were performed using a DSC 8230L (Rigaku, Japan) and thermogravimetry (TG) measurements were performed using a TG-DTA 9320 (Rigaku, Japan). Approximately 2–3 mg of sample for DSC and 15 mg for TG were accurately weighed into aluminum pans. The sample pans were heated at a rate of 3 °C min^−1^ from 25 to 250 °C and an empty aluminum pan was used as reference. Closed and open pans were used for DSC and TG measurements, respectively. 

#### 2.2.4. Single-Crystal X-Ray Diffraction and Refinements

Single-crystal X-ray diffraction patterns were recorded at 93 K in ω-scan mode (R-AXIS RAPID II, Rigaku, Japan) using a Cu-*K*α X-ray source (50 kV, 100 mA) and a graphite monochromator. The integrated and scaled data were empirically corrected for absorption effects using ABSCOR [39] and the initial structure was solved with SIR 2014 [40] using the direct method and then refined on *F*_o_^2^ with SHELXL 2014 [41]. All non-hydrogen atoms were anisotropically refined and hydrogen atom positions were calculated geometrically and included in the calculation using the riding model. Hydrogen atoms attached to nitrogen atoms were located using a differential Fourier map. All hydrogen atoms were freely refined and molecular graphics produced using Mercury 3.7 software [42].

#### 2.2.5. Solubility and Intrinsic Dissolution Rate

Each solid was dissolved in 2 mL of pH 1.2 solution and solubilities were determined after a 24-h incubation at 37 °C. During these measurements, a test tube was charged with excess solid and shaken using an orbital shaker (Miyamoto Riken, Tokyo, Japan) at a frequency of 120 Hz with the non-dissolved solids separated by filtration through a 0.45 µm membrane (Advantec, Tokyo, Japan). The intrinsic dissolution rate was determined using a Franz cell (Miyamoto Riken). All the samples were tableted into 1 cm diameter disks at 1.96 × 10^4^ N cm^−2^ and dissolution was carried out by placing these disks at the top of a vessel containing solvent at 37 °C and stirring at 300 rpm. Subsequently, 1 mL aliquots were drawn at specific times (0, 1, 3, 5, 7, 10, 20, 30, and 40 min) and filtered through a 0.45 μm ethyl cellulose filter (Advantec) using a glass syringe. The concentration of the drug in solution was calculated from its absorption at 230 nm for BEX-HCL cyclamate salt, and 277 nm for the saccharinate salt (V-370 UV–Vis spectrophotometer, Jasco, Tokyo, Japan) using a calibration curve.

#### 2.2.6. Dynamic Vapor Sorption 

Vapor sorption isotherms were measured by dynamic vapor sorption (DVS) at 25 °C using a Dynamic Vapor Sorption Advantage instrument (SMS Ltd., London, UK). Sieved samples were mounted on a balance and relative humidity (RH) was increased from 0 to 80% with 5% steps. The waiting time for 0.001% weight change was set to 15 min and the further step was increased or decreased automatically.

## 3. Results and Discussion

**Figure 1:**
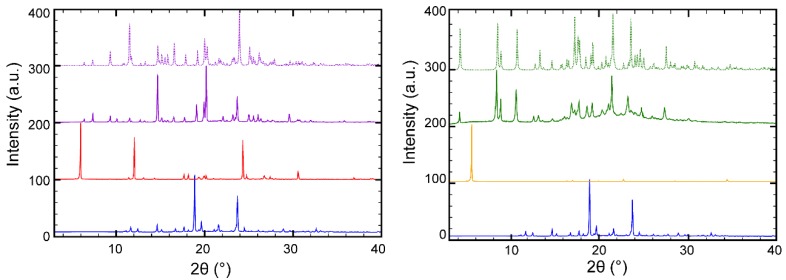
Powder X-ray diffraction (PXRD) patterns of benexate hydrochloride (BEX–HCl) (blue), sodium saccharinate (red), benexate-saccharinate (BEX–SAC) (purple), sodium cyclamate (yellow), and benexate-cyclamate (BEX–CYM) (green). The solid and dashed lines represent the experimental and simulated PXRD patterns, respectively.

The preparation of the novel salts presented in this study was achieved by an anion exchange reaction between chloride anion and saccharinate or cyclamate anion; therefore, the sodium salts of those sweeteners were chosen. Notably, other co-formers in either salt or acidic form failed to form new solid forms. As illustrated in Figure 1, there was no trace of raw material observed using this method, indicating the formation of new phases. Benexate-saccharinate (BEX–SAC) and benexate-cyclamate (BEX–CYM) were produced with sodium chloride as a byproduct of the anion exchange reaction. However, BEX–SAC and BEX–CYM could be easily separated from sodium chloride by filtration since those salts precipitated first and sodium chloride was retained in solution. This method was effective in producing pure BEX–SAC and BEX–CYM with high yield (>95%). The purities of BEX-SAC and BEX–CYM salts were confirmed by the good agreement between experimental and simulated PXRD patterns and the absence of raw material peaks. The difference in the experimental and simulated PXRD patterns was acceptable due to the preferred orientation effect. The anion exchange reaction also produced pure salts with a high yield of metformin-acesulfame salt [43]. Other attempts using high salt formation screening methods, such as slurry, co-grinding, dry milling using the same salt co-former and solvent system, failed, resulting in sticky amorphous solids.

**Figure 2:**
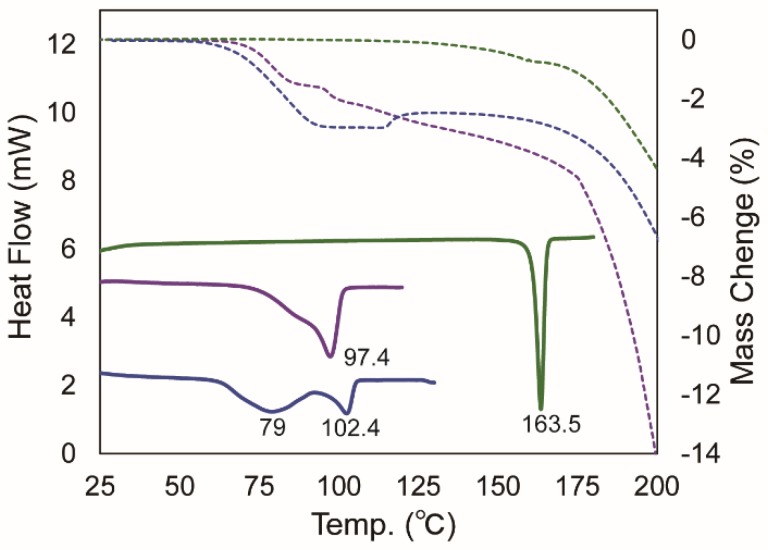
Differential scanning calorimetry (DSC) and thermogravimetry (TG) thermograms of BEX–HCl (blue), BEX–SAC (purple), and BEX–CYM (green). The solid and dashed lines represent DSC and TG thermograms, respectively.

To clarify the molecular entity produced after the anion exchange reaction, thermal analyses by means of DSC and TG measurements were performed (Figure 2). A comparison of the original BEX was difficult because of the hygroscopic and unstable nature of this compound. Therefore, a comparison of physicochemical properties presented in this study refer to BEX–HCl, which is the marketed form of BEX. A broad endothermic peak in the DSC thermogram of BEX-HCL was observed and the highest onset of this peak was found at 79.0 °C, indicating a desolvation/dehydration process. The dehydration process was subsequently followed by melting at 102.4 °C. The total mass loss corresponding to desolvation/dehydration was 4.01%, which was equivalent to one water molecule. A broad endothermic peak resembling desolvation/dehydration was also observed in the DSC thermogram of BEX–SAC, which overlapped with the melting process. The highest onset of the melting point of BEX–SAC was found to be 97.4 °C. The total mass loss corresponding to desolvation/dehydration was 2.92%. Considering the solvent used during the crystallization of BEX–SAC, it was likely that BEX–SAC existed as a monohydrate crystal. In contrast to BEX–HCL and BEX–SAC, no thermal moments prior to the melting process were observed with BEX–CYM, indicating that this crystal was either ansolvate or anhydrous. The highest onset of the melting point of BEX–CYM was found to be 163.5 °C. A significant mass loss was observed after the melting point in all crystals, indicating decomposition.

**Figure 3:**
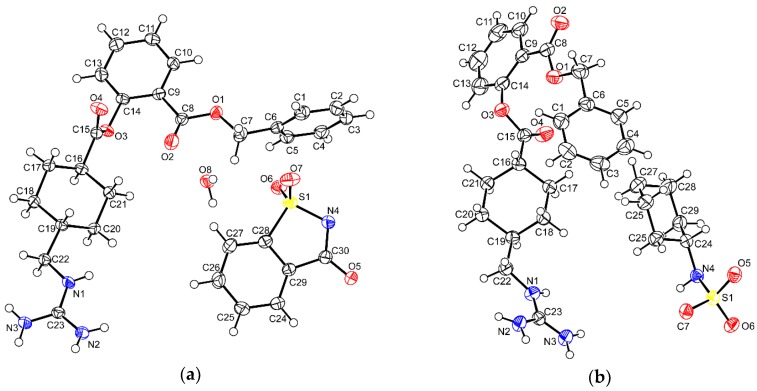
Thermal ellipsoid figures of (**a**) BEX–SAC and (**b**) BEX–CYM are drawn at the 50% probability level. The asymmetric unit of BEX–SAC contained a cationic benexate, an anionic saccharinate, and a water molecule. The asymmetric unit of BEX–CYM contained one cationic benexate molecule and one anionic cyclamate molecule.

Single-crystal X-ray crystallography is the most advanced technique for determining the structure of drug molecules. After the anion exchange reaction for preparing BEX–SAC and BEX–CYM, we were able to isolate single crystals from the reaction flask and perform single-crystal X-ray structure analysis of these crystals. The obtained crystallographic data are listed in Table 1 and reveal that both BEX–SAC and BEX–CYM belong to the triclinic crystal system and space group *P*-1. As shown in Figure 3, the asymmetric unit of BEX–SAC contained one cationic benexate, one anionic saccharinate, and one water molecule. The asymmetric unit of BEX–CYM contained one cationic benexate and one anionic cyclamate molecule. These results were in accordance with the thermal analysis wherein BEX–SAC and BEX–CYM existed as monohydrate and ansolvate/anhydrous crystals, respectively. All hydrogen atoms on N2 and N3 in both crystals were found in the residual density maps, and there was no significant residual density on N4 of the BEX–SAC crystal, which confirmed the saline nature of BEX–SAC and BEX–CYM.

The saline nature of BEX–SAC and BEX–CYM was not unexpected in this case. The p*K*_a_ of benexate was found to be 13.71. The p*K*_a_ values of saccharine and cyclamate as salt co-formers are 1.60 and −0.83, respectively. As the differences in p*K*_a_ were large enough—more than thrice the threshold value of 3 units—proton transfer was likely to have occurred in both crystals.

**Figure 4:**
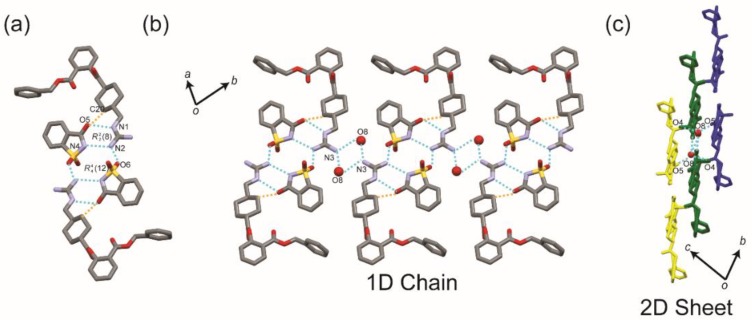
The hydrogen bond architecture in the BEX–SAC crystal. (**a**) The interaction involving two sets of cationic benexate and anionic saccharinate molecules and water constructs. (**b**) 1D chain structures parallel to the (110) plane. Each 1D chain (represented by different colors) interacts with each other to form (**c**) a 2D sheet structure. Conventional and non-conventional hydrogen bonds are drawn by dashed blue and orange lines, respectively. Hydrogen atoms have been omitted for clarity. The oxygen atoms from water molecules are drawn in ball-setting.

The crystal structure of BEX–SAC was constructed of complicated hydrogen bonds, as illustrated in Figure 4. One set of cationic benexate and anionic saccharinate were bound by a charged-assisted hydrogen bond N2^+^–H···N4^−^ and conventional hydrogen bond N1–H···O5, which enclosed the $R_{2}^{2}\left(8\right)$ hydrogen bond loop. An additional weak hydrogen bond C20–H···O5 was found to stabilize this interaction. An N2–H···O6 hydrogen bond connected two cationic benexates and anionic saccharinates. This hydrogen bond constructed an $R_{4}^{4}\left(20\right)$ loop around the center of symmetry, as shown in Figure 4a. Figure 4b illustrates that a one-dimensional (1D) chain structure was built by connecting each of the two sets of cationic benexates and anionic saccharinates along the (110) plane, which involved water molecules. The N3–H···O8 hydrogen bonds with two different symmetric operations (*x* + 1, *y*, *z* + 1 and –*x* + 2, −*y*, −*z* + 1) played important roles in building this 1D chain structure. 1D chains were connected to each other via O8–H···O4 and O8–H···O5 hydrogen bonds to establish a two-dimensional (2D) sheet structure, as illustrated in Figure 4c.

As explained above, the role of water molecules was crucial in constructing the 1D chain and 2D sheet structures in BEX–SAC crystals. Once the water molecules were expelled from the crystal, i.e., by heating, the crystal architecture collapsed, as expected, producing an anhydrous phase. This anhydrous phase was unstable and melted soon when heated further. Therefore, it was not surprising that the melting point of BEX–SAC was found to be relatively low (97.4 °C). This phenomenon was confirmed by the thermal analysis of BEX–SAC, which showed that dehydration and melting occurred simultaneously, without a specific range of temperature for producing the anhydrous phase.

**Figure 5:**
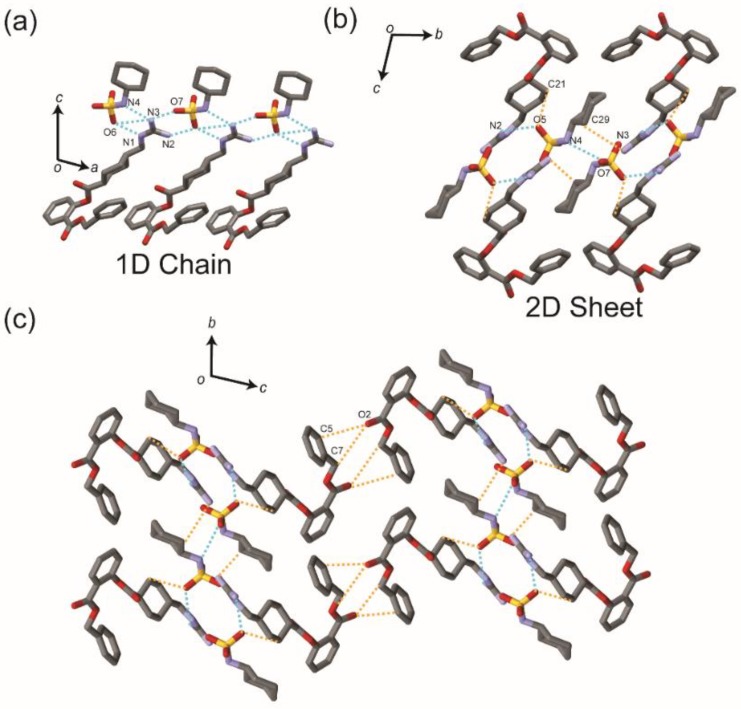
(**a**) 1D chain and (**b**) 2D sheet structures in the BEX–CYM crystal. (**c**) The 2D sheet structure is stacked along the *a*-axis through C5–H···O2 and C7–H···O2 hydrogen bonds. Conventional and non-conventional hydrogen bonds are drawn by dashed blue and orange lines, respectively. Hydrogen atoms have been omitted for clarity.

The crystal structure of BEX–CYM also contained complicated hydrogen bond networks. Conventional (N–H···N and N–H···O) and unconventional (C–H···O and C–H···N) hydrogen bonds were observed in the crystal. The N3 atom of guanidine from the benexate molecule formed three hydrogen bonds of N3–H3A···N4, N3–H3A···O6, and N3–H3B···O7, including bifurcate hydrogen bonds. The other N atoms of the guanidine moiety from the benexate molecule also interacted with O6 from the cyclamate anion to form N1–H···O6 and N2–H···O6 hydrogen bonds via –*x* + 1, −*y* + 1, −*z* + 1, and –*x* + 2, −*y* + 1, −*z* + 1 symmetry operations. These hydrogen bonds formed a 1D chain structure along the *b*-axis (Figure 5a). The hydrogen bonds N2–H···O5 and N4–H···O7 along with weak hydrogen bonds C29–H···N3 and C21–H···O5 connected the 1D chain to an adjacent 1D chain. This is hereafter defined as a 2D sheet structure (Figure 5b). These 2D sheet structures were stacked through C5–H···O2 and C7–H···O2 hydrogen bonds along the *a*-axis, as illustrated in Figure 5c.

**Figure 6:**
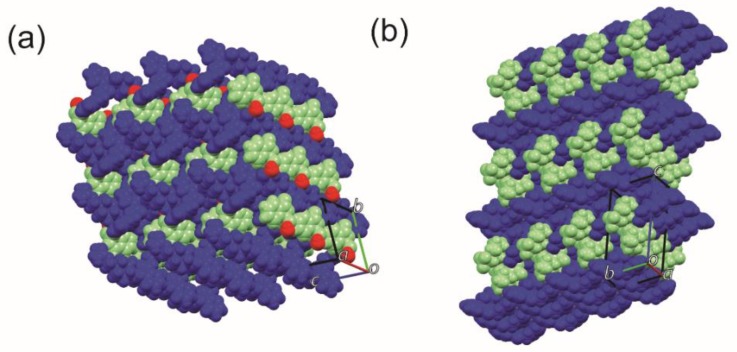
Packing view of (**a**) BEX–SAC and (**b**) BEX–CYM. All molecules are drawn in a space-fill setting. Cationic benexate, anionic salt co-former, and water molecules are represented by dark blue, light green, and red molecules, respectively.

Despite the hydrogen bond network differences between BEX–SAC and BEX–CYM, these crystals shared something in common. As shown in Figure 6, the packing motifs of BEX–SAC and BEX–CYM showed local layered-like structures composed of alternate arrangements between cationic benexate molecules and co-former molecules. Water molecules were also involved in the formation of a local layered-like structure in BEX–SAC.

The preparation of new solid forms is an effective way to change the physicochemical properties of drugs that have problematic physicochemical properties. Considering the fact that BEX and BEX–HCl have low solubility in aqueous media, we performed solubility and dissolution rate measurements. The solubility measurements here are expected to represent the thermodynamic aspect of the solid–liquid interface, while the latter represents the kinetic aspect. The dissolution measurement presented in this study was performed with a Franz cell (non-United Stated Pharmacopoeia method) to get pre-concentrated samples owing to the insoluble nature of BEX–HCL. 

**Figure 7:**
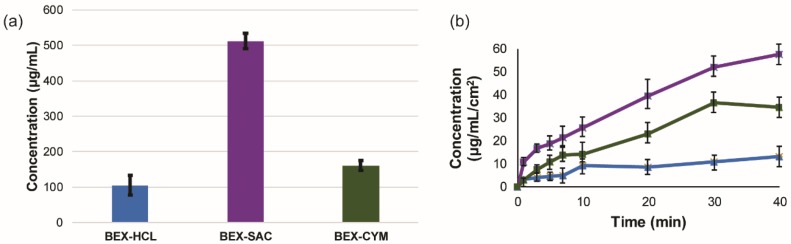
(**a**) The solubility and (**b**) intrinsic dissolution rate of BEX–HCl (blue), BEX–SAC (purple), and BEX–CYM (green). Solubility and dissolution rate experiments were conducted in triplicate.

The solubility of BEX–HCL was found to be 104.42 ± 27.60 µg/mL. All new solid forms presented in this study showed improved solubility (Figure 7a). The solubility of BEX-SAC and BEX–CYM was 512.16 ± 22.06 and 160.53 ± 14.52 µg/mL, respectively. This means that the solubility increased 5 and 1.5 times relative to the marketed form of BEX. In agreement with the solubility results, the intrinsic dissolution rate also showed the same trend with BEX–SAC and BEX–CYM showing improved dissolution rates of around 5 and 2 times, respectively (Figure 7b). All solid forms remained the same at the end of the solubility and dissolution rate experiments, as confirmed by PXRD measurements.

We tried to rationalize the solubility and dissolution rate improvement by considering the molecular arrangement in the crystal structure. As mentioned above, the overall packing features in BEX–SAC and BEX–CYM showed local layered-like structures composed of an alternate arrangement between cationic benexate molecules and co-former molecules. This local layered-like structure facilitated structural collapse during dissolution by propagation interaction loss of the drug and salt co-former. This mechanism has been proposed in studies aimed at improving the solubility of insoluble drugs [16,20,21,22,23]. Given the fact that higher solubility and improved dissolution rate profiles were observed with the novel salts of BEX, it is likely that an improvement in the bioavailability of BEX could be observed if these solid forms are chosen. At a minimum, the results presented in this work provide a fundamental step for the further development of BEX, i.e., pharmacokinetics and stability studies.

**Figure 8:**
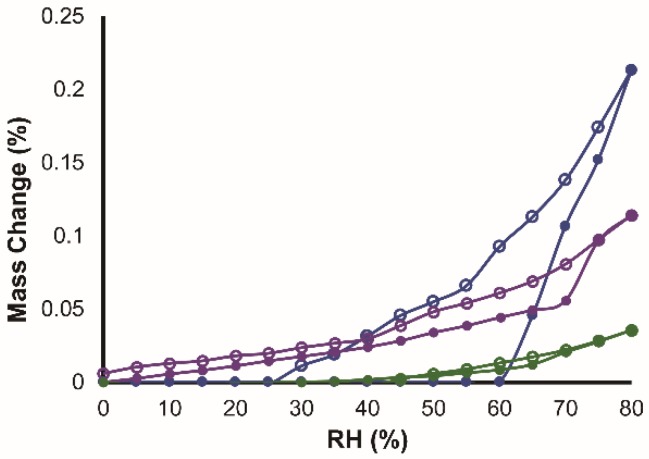
Water sorption–desorption of BEX–HCl (blue), BEX–SAC (purple), and BEX–CYM (green). The closed and open symbols represent sorption and desorption, respectively.

In addition to the solubility and the dissolution rate, hygroscopicity is also an important physicochemical property of pharmaceutical materials. Hygroscopicity was measured using a water sorption–desorption isotherm. As shown in Figure 8, BEX–HCl was slightly hygroscopic because the water uptake at 25 °C/80 RH was 0.213% (*w*/*w*). The total water uptake at 25 °C/80 RH of BEX–SAC and BEX–CYM were 0.114 and 0.035% (*w*/*w*), respectively. Therefore, in this case, BEX–SAC and BEX–CYM were categorized as non-hygroscopic. The strongest resistance to moisture uptake was displayed by BEX–SAC. The tendency to dehydrate was not observed in BEX–HCL and BEX–SAC although both these crystals were hydrate crystals, as indicated by the step-change in the sorption–desorption profile. All crystalline forms of BEX–HCl, BEX–SAC, and BEX–CYM remained unchanged, as attested by PXRD analysis.

## 4. Conclusions

Novel salts of benexate with artificial sweeteners of saccharine and cyclamate were prepared using the anion exchange reaction method. The solid–state properties of these new crystals were measured by PXRD, DSC, and TG and compared to benexate hydrochloride monohydrate, the marketed form of benexate. We also succeeded in solving the crystal structures of these novel salts using single-crystal X-ray crystallography. Benexate saccharinate monohydrate and benexate cyclamate showed higher solubilities and faster dissolution profiles than benexate hydrochloride monohydrate. A local layered structure was proposed to be responsible for these improvements. The new salts also showed better resistance against moisture uptake and were categorized as non-hygroscopic. The results presented in this study suggest the feasibility of developing novel salts of benexate with better physicochemical properties.

## Figures and Tables

**Table 1:** Crystallographic details of benexate-saccharinate (BEX–SAC) and benexate-cyclamate (BEX–CYM).

Parameters	BEX–SAC	BEX–CYM
Moiety formula	C_23_H_28_N_3_O_4_·C_7_H_4_NO_3_S·H_2_O	C_23_H_28_N_3_O_4_·C_6_H_12_NO_3_S
Formula weight	610.67	588.71
Crystal system	Triclinic	Triclinic
Space group	*P*-1	*P*-1
*a* (Å)	8.8182 (2)	6.97200 (18)
*b* (Å)	12.8387 (3)	10.3577 (3)
*c* (Å)	14.1237 (4)	21.5218 (6)
*α* (°)	82.167 (6)	103.718 (7)
*β* (°)	79.787 (6)	91.536 (6)
*γ* (°)	70.139 (5)	96.783 (7)
*V* (Å^3^)	1475.06 (8)	1496.85 (9)
*Z, Z*′	2, 1	2, 1
*T* (K)	173	173
Unique ref.	5291 (*R*_int_ = 0.031)	5377 (*R*_int_ = 0.031)
Refined parameters	416	394
Goodness-of-fit on *F*^2^	1.06	1.13
Final *R* indices [*I* > *2σ* (*I*)]	*R*_1_ = 0.0337	*R*_1_ = 0.0418
CCDC number	1840914	1840913

## Supplementary Materials

CCDC 1840913 and 1840914 contain the supplementary crystallographic data for this paper. These data can be obtained free of charge via www.ccdc.cam.ac.uk/data_request/cif or by emailing data_request@ccdc.cam.ac.uk or by contacting The Cambridge Crystallography Data Centre, 12 Unior Road, Cambridge CB2 1EZ, UK; fax: +44 1223 336033. The following is available online at http://www.mdpi.com/1999-4923/10/2/64/s1, Figure S1: PXRD patterns of BEX-SAC (blue) BEX-CYM (red) before and after dissolution experiments.
