# Supplementary material for: Solubility Improvement of Benexate through Salt Formation Using Artificial Sweetener
Source: Pharmaceutics. 2018 May 26;10(2):64. doi: 10.3390/pharmaceutics10020064 (PMC6027452; doi:10.3390/pharmaceutics10020064)
Supplement: Supplementary file 1 [file pharmaceutics-10-00064-s001.zip › supplementary.docx]

Supplementary Materials

Solubility Improvement of Benexate through Salt Formation Using Artificial Sweetener

Okky Dwichandra Putra ^1,2,#^, Daiki Umeda ^1^, Eriko Fujita ^1^, Tamami Haraguchi ^3^, Takahiro Uchida ^3^, Etsuo Yonemochi ^1,^*, and Hidehiro Uekusa ^2,^*

^1^ School of Pharmacy and Pharmaceutical Sciences, Hoshi University, 2-4-41 Ebara, Shinagawa, Tokyo 142-8501, Japan.

^2^ Department of Chemistry, Tokyo Institute of Technology, 2-12-1 Ookayama, Meguro, Tokyo 152-8551, Japan.

^3^ Faculty of Pharmaceutical Sciences, Mukogawa Women´s University, 11-68 Koshien 9-Bancho, Nishinomiya, Hyogo 663-8179, Japan.

^#^ Current Address: Pharmaceutical Technology and Development, AstraZeneca Gothenburg, Pepparedsleden 1, Mölndal S-413 38, Sweden.

***** Correspondence: [e-yonemochi@hoshi.ac.jp](mailto:e-yonemochi@hoshi.ac.jp) (E.Y.) and [uekusa@chem.titech.ac.jp](mailto:uekusa@chem.titech.ac.jp) (H.U.)


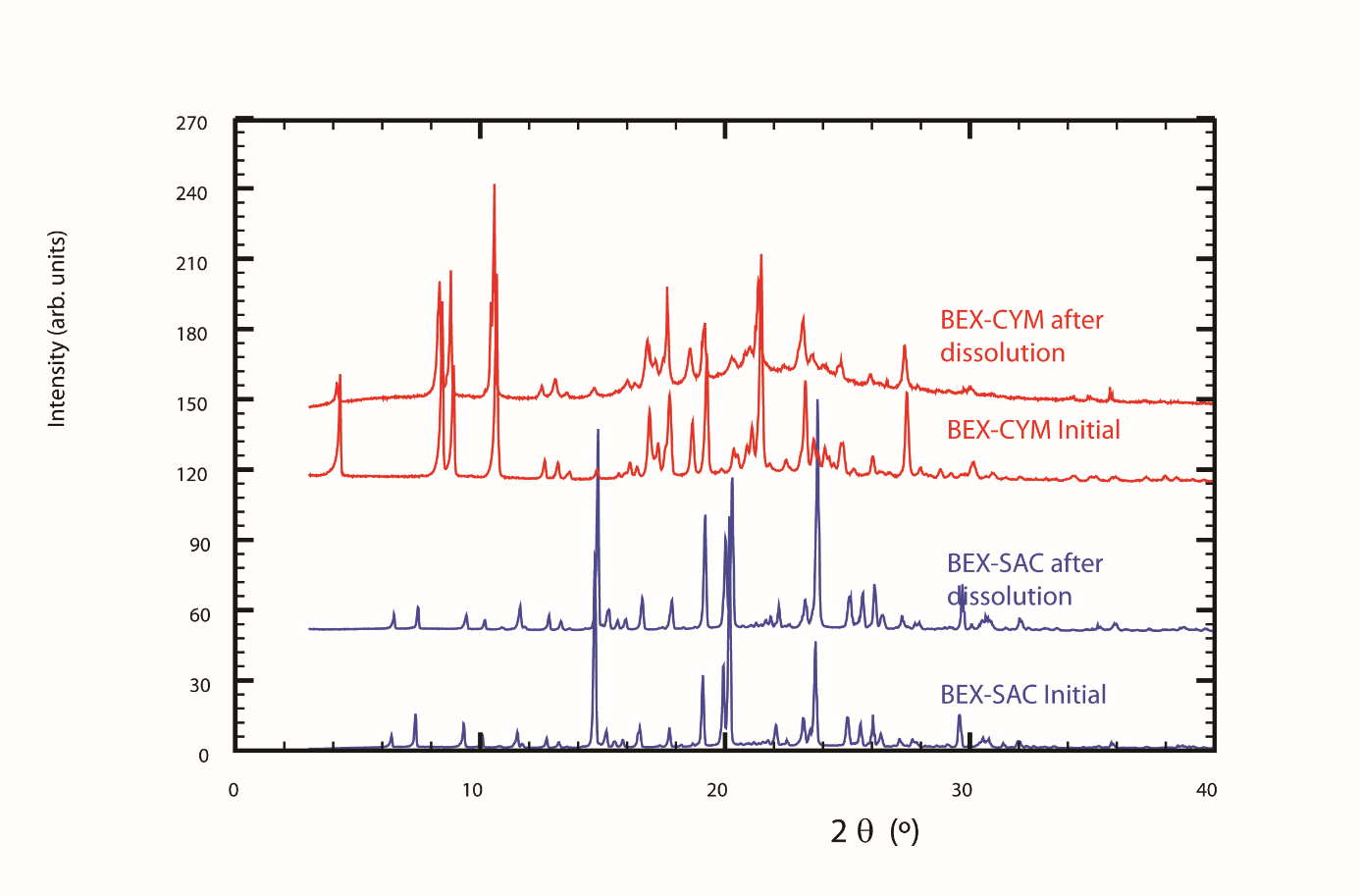


**Figure S1.** PXRD patterns of BEX-SAC (blue) BEX-CYM (red) before and after dissolution experiments.
